# Acute Tension Pneumothorax Following Cardiac Herniation after Pneumonectomy

**DOI:** 10.1155/2010/213818

**Published:** 2010-06-14

**Authors:** Daniel Steinmann, Eva Rohr, Andreas Kirschbaum

**Affiliations:** ^1^Department of Anaesthesia and Critical Care Medicine, University Hospital Freiburg, Hugstetter Straße 55, 79106 Freiburg, Germany; ^2^Department of Thoracic Surgery, University Hospital Freiburg, Hugstetter Straße 55, 79106 Freiburg, Germany

## Abstract

A tension pneumothorax is one of the main causes of cardiac arrest in the initial postoperative period after thoracic surgery. Tension pneumothorax and cardiac herniation must be taken into account in hemodynamically unstable patients after pneumonectomy. We report an unusual case of successful treatment of acute tension pneumothorax following cardiac herniation and intrathoracic bleeding after pneumonectomy.

## 1. Introduction

Following thoracic surgery, a tension pneumothorax is one of the main causes of cardiac arrest in the initial postoperative period [1]. Tension pneumothorax and cardiac herniation are serious complications in hemodynamically unstable patients after pneumonectomy, especially in cases where a pericardial flap for reinforcement of the bronchial stump is used [2–4]. Immediate diagnosis and appropriate treatment in such situations is crucial [5]. We report an unusual postoperative course of a patient with acute tension pneumothorax following cardiac herniation and intrathoracic bleeding after pneumonectomy.

## 2. Case Report

**Figure 1:**
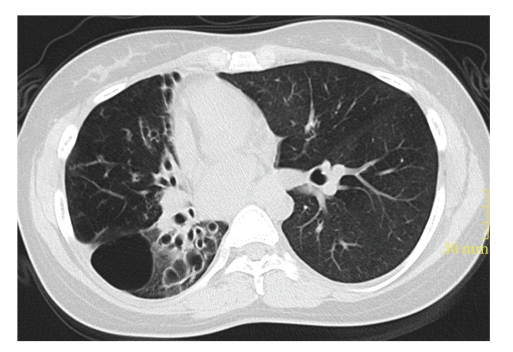
Preoperative chest CT scan revealing right accentuated severe bronchiectasis accompanied by pulmonary emphysema.

The patient, a 34-year-old woman (height 156 cm, weight 46 kg), showed symptoms of chronic cough, recurrent pulmonary infections, and pronounced stress dyspnea. Further evaluations revealed tuberculosis, treated several years with an unknown monotherapy. In the respiratory function test, forced vital capacity was 1.6 L (FVC 52%) and forced expiratory volume in 1 second was 0.91 L (FEV1 33.5%), indicating marked restricted lung function. A chest CT scan revealed right accentuated severe bronchiectasis accompanied by pulmonary emphysema (Figure 1). During a rigid bronchoscopy, a damage of the right main stem bronchus and massive purulent secretions of the right caudal lung sections (affected by Escherichia coli) were found. Lung ventilation-perfusion scintigraphy showed a distribution of ventilation of 90% on the left and 10% on the right side. Thus, decision for right pneumonectomy was made.

**Figure 2:**
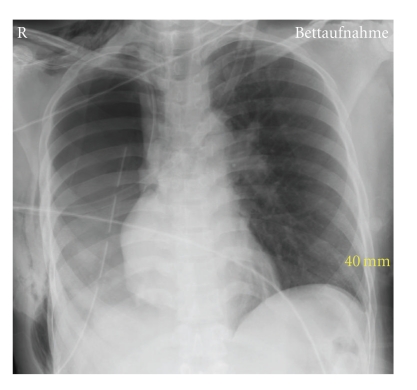
Chest X-rays of the patient immediately after pneumonectomy.

Induction and maintenance of anesthesia was uneventful. The patient received a left-sided double-lumen tube for one-lung ventilation, a central venous catheter in the right subclavian vein, and a radial arterial line. A thoracic epidural catheter was inserted for perioperative pain control. Pneumonectomy was performed through a lateral thoracotomy. To reduce the risk of postoperative bronchial stump dehiscence, the bronchial stump was covered with a pedicled pericardial flap, and the pericardial defect was immediately repaired with a Vicryl mesh. A chest tube was placed in the right cavity. At the end of surgery the patient was successfully extubated and transferred to the intensive care unit. The postoperative chest X-ray showed correct position of the central venous catheter and the chest tube (Figure 2).

**Figure 3:**
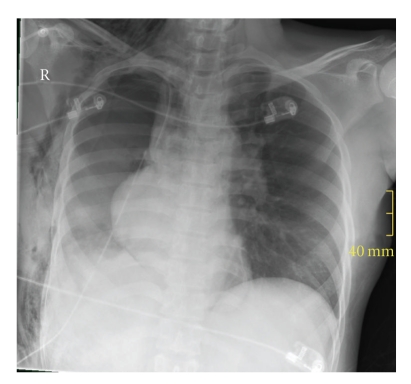
Chest X-rays of the patient with cardiac herniation to the right side.

A few hours later, the patient became hemodynamically unstable and showed an anemia (Hb: 6.7 g/dl). Because of an assumed intrathoracic hemorrhage and possible cardiac herniation (Figure 3), the patient was taken back to the operating room. On reopening of the thoracotomy, cardiac herniation into the right thoracic cavity because of a rupture of the Vicryl mesh was seen. A concomitant rupture of the right lower pulmonary vein and the left atrium was also observed. Surgical treatment included suture of the pulmonary vein and left atrium as well as repair of the pericardium with a Gore-Tex patch. Volume substitution for blood loss consisted of 1.500 lactated Ringer's solution, 1000 ml of 6% hydroxyethylstarch, and 600 ml packed red cells elevating hemoglobin to 10 g/dl.

**Figure 4:**
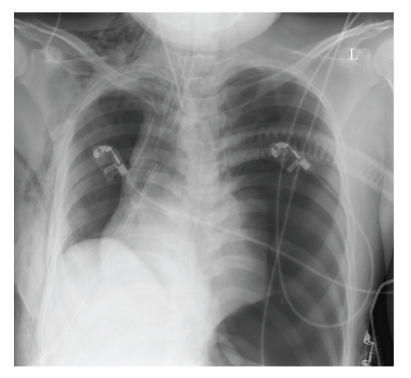
Chest X-rays of the patient showing a tension pneumothorax on the left side with mediastinal shift.

**Figure 5:**
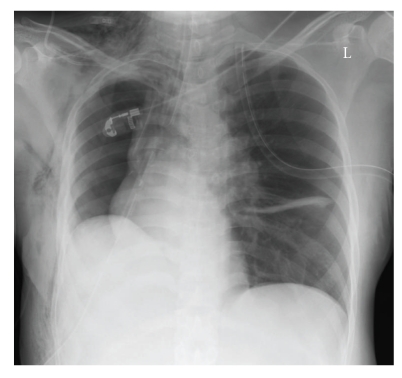
Chest X-rays of the patient after insertion of a chest tube and re-expansion of the left lung.

During transfer of the patient to the intensive care unit after surgery, ventilation became increasingly difficult and again hemodynamical instability occurred. Carotid pulse was missing and invasive measured blood pressure displayed 39/31 mmHg. The patient received ephedrine (100 mcg) elevating blood pressure to 90/60 mmHg. A chest x-ray was made (Figure 4) which revealed an acute tension pneumothorax on the left side with mediastinal shift. An immediate needle decompression was attempted with a 16 G cannula at the left second intercostal space in the midclavicular line. The tension pneumothorax was successfully relieved and an intercostals tube (24 F) was inserted in the left fourth intercostal space in the midaxillary line (Figure 5). Following treatment of the tension pneumothorax, ventilation and cardiac output quickly normalized. The patient was uneventfully extubated 8 h later and discharged from the intensive care unit in stable condition on the third postoperative day. The cause of the tension pneumothorax remained unknown.

## 3. Discussion

Pneumonectomy for inflammatory lung disease, bronchiectasis, tuberculosis, and other nonmalignant conditions are quite uncommon in modern-days medicine [6]. Despite many efforts, pneumonectomy remains a challenging operation, carrying many complications and anatomic and physiologic changes [6]. Bronchopleural fistula is a serious complication after pneumonectomy. Thus, bronchial stump reinforcement with a pericardial flap is used for prevention of bronchopleural fistula in selected patients [7]. The resulting defect in the pericardium can be reconstructed with Vicryl mesh with good success [7]. The potential side effects of such a procedure are arrhythmias in the postoperative period, infection of the foreign material, and cardiac tamponade in case of tight reconstruction [7]. In our patient, the initial used Vicryl mesh was not able to cover the defect in the pericardium resulting in cardiac herniation. Thus, we decided to use a Gore-Tex patch with an excellent postoperative result.

During the postoperative period after thoracic surgery several main causes of hemodynamical instability and cardiac arrest must be taken into consideration: hemorrhage causing hypovolemic shock, cardiac tamponade, cardiac herniation, and tension pneumothorax [1, 4, 5]. In patients undergoing pneumonectomy, particularly right pneumonectomy with pericardial resection or usage of a pericardial flapfor reinforcement of the bronchial stump, cardiac herniationhas been described [2–4]. In these cases and in our patient, an anterior-posterior chest X-ray was a useful diagnostic tool. Cardiac herniation should be treated by immediate re-thoracotomy and removal of the underlying cause for the herniation. Turning the patient on the unaffected side usually improves hemodynamic parameters during cardiac herniation and can bridge the time for transportation to the operating room [2–4].

Cardiac herniation may be caused by a tension pneumothorax after thoracotomy requiring insertion of a intercostals drainage and re-thoracotomy [1, 4, 5]. However, in our patient, the tension pneumothorax occurred after re-thoracotomy for treatment of a cardiac herniation, making diagnosis difficult. A tension pneumothorax should be rapidly diagnosed by clinical signs: difficulty with ventilation/respiratory distress, desaturation, hypotension, heart rate changes, unilateral chest expansion, abdominal distension, distended neck veins, raised CVP, and tracheal deviation [8]. If uncertainty exists, especially in the case of pneumonectomy, and circumstances permitting, a prompt chest X-ray is useful to confirm a tension pneumothorax. Appropriate treatment consists of decompression by needle thoracocentesis followed by the insertion of a chest tube [4, 5, 8].

In summary, we present the successful treatment of a patient with an acute tension pneumothorax following cardiac herniation and intrathoracic bleeding after pneumonectomy. Immediate re-thoracotomy and needle thoracocentesis are essential procedures in such circumstances and should be conducted without delay. We hope that our report will contribute to adequate postoperative care of patients with serious complications after pneumonectomy.

## References

[1] Soar J, Deakin CD, Nolan JP, Abbas G, Alfonzo A, Handley AJ, Lockey D, Perkins GD, Thies K (2005). European Resuscitation Council Guidelines for Resuscitation 2005: section 7. Cardiac arrest in special circumstances. *Resuscitation*.

[2] Veronesi G, Spaggiari L, Solli PG, Pastorino U (2001). Cardiac dislocation after extended pneumonectomy with pericardioplasty. *European Journal of Cardio-Thoracic Surgery*.

[3] Shimizu J, Ishida Y, Hirano Y, Tatsuzawa Y, Kawaura Y, Nozawa A, Yamada K, Oda M (2003). Cardiac herniation following intrapericardial pneumonectomy with partial pericardiectomy for advanced lung cancer. *Annals of Thoracic and Cardiovascular Surgery*.

[4] Sasidharan B, Moideen I, Warrier G, Prabhu A, Koshy S, Nair SG, Rao SG, Shivaprakasha K (2006). Cardiac herniation following closure of atrial septal defect through limited posterior thoracotomy. *Interactive Cardiovascular and Thoracic Surgery*.

[5] Nolan JP, Deakin CD, Soar J, Böttiger BW, Smith G (2005). European Resuscitation Council Guidelines for Resuscitation 2005: section 4. Adult advanced life support. *Resuscitation*.

[6] Fuentes PA (2003). Pneumonectomy: historical perspective and prospective insight. *European Journal of Cardio-TSurgery*.

[7] Taghavi S, Marta GM, Lang G, Seebacher G, Winkler G, Schmid K, Klepetko W (2005). Bronchial stump coverage with a pedicled pericardial flap: an effective method for prevention of postpneumonectomy bronchopleural fistula. *Annals of Thoracic Surgery*.

[8] Bacon AK, Paix AD, Williamson JA, Webb RK, Chapman MJ (2005). Crisis management during anaesthesia: pneumothorax. *Quality &amp; Safety in Health Care*.

